# “But it feels swollen!”: the frequency and clinical characteristics of people with knee osteoarthritis who report subjective knee swelling in the absence of objective swelling

**DOI:** 10.1097/PR9.0000000000000971

**Published:** 2021-11-08

**Authors:** So Tanaka, Tomohiko Nishigami, Koji Ohishi, Kazutaka Nishikawa, Benedict M. Wand, Tasha R. Stanton, Hirofumi Yamashita, Akira Mibu, Masami Tokunaga, Takaaki Yoshimoto, Takahiro Ushida

**Affiliations:** a Department of Rehabilitation, Fukuoka Orthopaedic Hospital, Fukuoka, Japan; b Department of Unifying Pain Medicine, Graduate School of Medicine, Aichi Medical University, Nagakute, Japan; c Faculty of Health and Welfare, Department of Physical Therapy, Prefectural University of Hiroshima, Mihara, Japan; d Department of Orthopaedic Surgery, Nishikawa Orthopaedic Clinic, Dazaifu, Japan; e The Faculty of Medicine, Nursing and Midwifery and Health Sciences, The University of Notre Dame Australia, Fremantle, WA, Australia; f IIMPACT in Health, Allied Health & Human Performance Academic Unit, The University of South Australia, Adelaide, SA, Australia; g Department of Rehabilitation, Nozomi Orthopaedic Clinic Saijo, Higashihiroshima, Japan; h Department of Physical Therapy, Konan Women's University, Kobe, Japan; i Department of Orthopaedic Surgery, Fukuoka Orthopaedic Hospital, Fukuoka, Japan; j Multidisciplinary Pain Center, Aichi Medical University, Nagakute, Japan

**Keywords:** Knee, Osteoarthritis, Swelling, Body perception, Ultrasonography

## Abstract

**Introduction:**

There are complex interactions between pain and perceptions of the painful body part in musculoskeletal disorders, and disruption of various body representations in people with chronic pain.

**Objectives:**

The purpose of this study was to investigate how frequently people with knee osteoarthritis (OA) complain of swelling without objective evidence of swelling, and describe the clinical characteristics of this population.

**Methods:**

Forty-six people with knee OA (68.1 ± 8.8 years) participated in this cross-sectional study. Subjective and objective swelling was evaluated by knee-specific body perception questionnaire and ultrasonography, respectively. Pain intensity, disability, pain-related beliefs, 2-point discrimination threshold, and quadriceps muscle strength were also evaluated.

**Results:**

Approximately 1/3 of participants (n = 15) had subjective feelings of knee swelling in the absence of objective swelling (S only). Fifteen participants had both subjective and objective knee swelling (S + O group) and 16 had neither subjective nor objective knee swelling (No S/O group). Participants in the S only group had similar pain or disability as those in the S + O group but had more severe pain or disability than those with in the No S/O group. Those in the S only group also had larger 2-point discrimination distance threshold at the medial knee (impaired tactile acuity) than those in the S + O group and had more dysfunctional pain catastrophizing and pain-related self-efficacy than both other groups.

**Conclusion:**

Our results suggest that about 30% of people with knee OA perceive swelling of the knee in the absence of any objective swelling and that this is accompanied by severe pain and functional disability. Considering altered body image of the knee may reveal relevant treatment-based subgroups in people with knee OA.

## Abstract 2

Supplemental Digital Content is Available in the Text.

The present results support the coexistence of altered body image (subjective without objective swelling) and pain, disability, and maladaptive beliefs in people with knee osteoarthritis.

## 1. Introduction

Data from multiple sources suggest that there are complex interactions between pain and perceptions of the painful body part in musculoskeletal disorders,^52,56^ and numerous studies have revealed disruption of various body representations in people with chronic pain.^45^ One consistent finding is that people with pain often report that the painful area feels enlarged or swollen.^21,29,34,41^ Furthermore, although experimental pain studies involving noxious stimulation to the skin provide mixed results,^25,60^ visual illusions that magnify the size of the body part have been shown to increase pain with movement in people with complex regional pain syndrome^31^ and delayed onset muscle soreness,^55^ suggesting a possible causal relationship between perceptions of enlargement and movement evoked pain.

Preliminary evidence indicates that disrupted body perception might be a feature of painful knee osteoarthritis (OA). The Fremantle Knee Awareness Questionnaire (FreKAQ) was developed to measure knee-specific body perception in people with knee pain^35^ by modifying the low back pain version,^36,58^ and higher scores on this scale are associated with higher levels of movement-evoked pain and knee pain–related disability in knee OA.^28,35^ In addition, higher FreKAQ scores at baseline were associated with failing to achieve clinically meaningful levels of pain reduction with a three-month evidence-based education and exercise programme.^54^ Furthermore, cluster analysis suggests that disrupted knee perception may explain some of the discrepancy between knee pain–related disability and severity of radiographic findings in knee OA.^37^ Item 7 of the FreKAQ (back translated from Japanese: I feel like my knee is bigger [swollen]), specifically asks about knee size and the difference between perception and reality. We found that this item was significantly easier to endorse for people with knee OA than the similarly worded item was for people with low back pain,^35^ neck pain,^63^ or shoulder pain,^38^ which suggests this issue might be particularly relevant in knee OA.

One potential reason why this item is more readily endorsed in knee OA is that knee swelling is relatively common in this condition.^9,12^ Currently it is unclear if the endorsement of this item reflects a truly enlarged (objectively swollen) knee or a knee that is simply perceived as enlarged. We were interested in exploring this concept, particularly the interaction between the perception of an enlarged knee and objective markers of swelling within the knee and its influence on clinical status. The aims of this study, therefore, were to investigate how common it is in people with knee OA to perceive their knee as enlarged without objective swelling being present. Furthermore, we aimed to describe the clinical characteristics of people who did and did not have perceived or objective knee swelling.

## 2. Methods

### 2.1. Study design

Ethical approval was obtained from the Institutional Ethics Committee of Nishikawa Orthopaedic Hospital (Approval number: 20181005). Written informed consent was obtained from all participants before the study. The study was conducted in compliance with the Declaration of Helsinki.

### 2.2. Setting and participants

Participants were consecutively sampled from an orthopaedic outpatient clinic between February 2019 and April 2020. People with symptomatic knee OA, diagnosed according to the American College of Rheumatology clinical or radiographic classification criteria,^2^ and aged between 40 and 85 years were considered for inclusion in this study. People were excluded if they had mechanical derangement of the knee (eg, meniscal lesion, loose body, or anterior cruciate ligament instability), any neurological disorder affecting lower-limb function, significant cognitive impairment, neurological or orthopedic injury that might affect touch discrimination at the knee, significant uncorrectable visual impairment, concomitant diagnosis of fibromyalgia, a psychiatric disorder, or had undergone previous knee surgery such as arthroplasty, arthroscopy, or osteotomy. All participants underwent an x-ray examination and were examined and screened for eligibility by an orthopedist (K.N.), who also confirmed the presence of current knee joint pain.

### 2.3. Measurement

The affected knee and unaffected knee were determined for each participant. In those with bilateral knee pain, the most painful knee was deemed the affected side. Where appropriate, we report data from both knees, but only data from the affected knee were used to compare between groups. Demographic data (age, gender, and body mass index), severity of degenerative changes, and the presence or absence of regular nonsteroidal anti-inflammatory drug use were assessed in all participants. Severity of osteoarthritic changes in the knee was evaluated using the Kellgren and Lawrence (K/L) grade, which is a method of classifying the severity of knee OA using a 5 point scale, with 0 representing no features of knee OA and IV severe sclerosis and bone deformity.^20^ All participants in the current study had a Kellgren–Lawrence (KL) score of II or more.

#### 2.3.1. Classification using subjective and objective measures of swelling of the knee joint

The degree of subjective swelling was assessed using item 7 of the FreKAQ (back translated from the Japanese “I feel like my knee is bigger [swollen]”).^35^ The FreKAQ has 5 response categories: “never,” “rarely,” “occasionally,” “often,” and “always.” We defined the patient as having subjective swelling if the patient answered “often” or “always” to this item.

**Figure 1.**
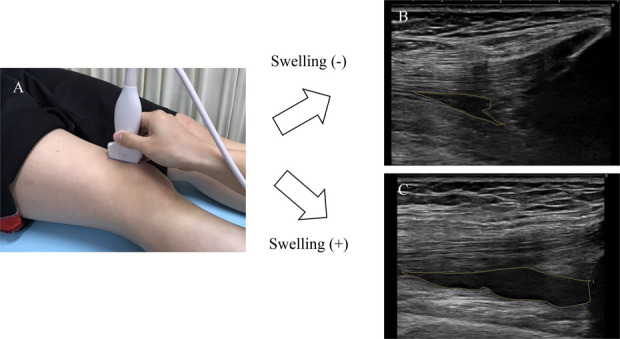
Quantitative evaluation of effusion in the suprapatellar bursa (objective swelling). (A) The ultrasound scan image of the suprapatellar bursa was acquired by placing a linear probe longitudinally on the suprapatellar pouch. During the examination, the transducers were placed as gently as possible, as pressure on the skin through the transducers can affect the acquired effusion area. Representative images of the suprapatellar swelling area: 30 mm^2^ (B); 100 mm^2^ (C).

The objective degree of swelling was evaluated using ultrasonography (10-MHz, SONIMAGE HS1 version 1.31; Konica Minolta, Inc, Tokyo, Japan) based on a previously published protocol.^6^ All ultrasound scans were evaluated by 2 independent examiners (S.T., who has 15 years of experience in musculoskeletal ultrasonography, and K.O., who has 10 years of experience). The ultrasound scan assessed the degree of effusion in the suprapatellar bursa. The subjects were examined lying in the supine position with both knees semiflexed to 20° and their feet in the neutral position. The ultrasound scan image of the suprapatellar bursa was acquired by placing a linear probe longitudinally on the suprapatellar pouch. During examination, the transducers were placed as gently as possible, as pressure on the skin through the transducers can affect the acquired effusion area. The effusion area was calculated by tracing the margin of the echo-free space that corresponds with the suprapatellar pouch. Although the margins of these echo-free spaces were traced, the area (mm^2^) of suprapatellar effusion was calculated automatically (Fig. 1). In accordance with previous studies, we defined the participant as having objective swelling if an echo-free area of 90 mm^2^ or more was present in the suprapatellar bursa,^6^ an approach that has high reliability.^5^

On the basis of the results of these 2 swelling measures, people were categorized into 3 groups: the subjective swelling only group (S only), the subjective and objective swelling group (S + O), and the no subjective or objective swelling group (No S/O). No participant who had objective evidence of knee swelling failed to perceive the knee as enlarged.

#### 2.3.2. Disturbed body perception

An overall measure of self-reported body perception of the affected knee was obtained using the total score derived from all items of the FreKAQ questionnaire.^35^ The FreKAQ is composed of 9 items that relate to neglect-like symptoms, reduced proprioceptive acuity, and perceived body part shape and size. Higher scores on the FreKAQ indicate more disturbed body perception.

#### 2.3.3. Pain intensity

Pain intensity at rest and during movement was measured for both knees using a 0 to 10 Numeric Rating Scale anchored at the left with “0 = no pain” and at the right with “10 = unbearable pain.” Pain with movement was evaluated in reference to the following question, “What is the intensity of your knee pain with movement?”

#### 2.3.4. Disability

Disability was measured using the Japanese-validated version of the Oxford Knee Score for people with knee OA.^10,53^ The scale is scored from 0 to 48, with higher scores indicating better function.

#### 2.3.5. Two-point discrimination thresholds

Two-point discrimination (TPD) thresholds were measured on both the medial and lateral sides of the knee. Tactile acuity, as measured by the TPD threshold, is considered a possible clinical signature of the primary sensory cortex representation of the tested area.^43^ Two-point discrimination was assessed using a digital Vernier caliper (Plastic LCD Digital Caliper, Duratech, La Crosse, WI) and was defined as the smallest distance between caliper points at which the participant could clearly detect 2 points instead of one. Following the Moberg protocol, this measure was tested in the vertical direction on the medial (2 cm medial of the medial border of the patella) and lateral (2 cm lateral of the lateral border of the patella) aspect of both knees, using the tibiofemoral joint line as a reference point.^50^ Data from one descending run and one ascending run at each location were averaged to obtain the final value.^27^

#### 2.3.6. Range of motion

The same investigator (S.T.) measured the range of motion (ROM) of both knee joints with a standard goniometer. Knee flexion ROM was the value of active bending of the knee while the patient was lying supine.^3^ Knee extension ROM was the angle of passive straightening of the knee while the patient was lying supine.^59^

#### 2.3.7. Quadriceps muscle strength

Maximal voluntary isometric knee extension strength was measured with the participant sitting,^13,39^ using a calibrated dynamometer (Micro FET 2; Hoggan Scientific, LLC, Salt Lake City, UT). The thigh was fixed to the seat at the distal femur. The moment arm was attached to the tibia just above the malleoli. The knee and hip angles were fixed at 90°. Both legs were tested separately, and the trial order was randomized. The subjects performed as many maximal actions until the peak value no longer increased. The results were divided by body weight and expressed as N·m/kg. The examiner assessing TPD thresholds, ROM, and strength was blind to swelling classification.

#### 2.3.8. Pain catastrophizing

Pain-related catastrophizing was measured using the Japanese version of the Pain Catastrophizing Scale.^26^ The scale comprises 13 items related to magnification, rumination, and helplessness about pain with higher scores indicating greater levels of pain-related catastrophization.^51^

#### 2.3.9. Pain self-efficacy

The Japanese version of the Pain Self-Efficacy Questionnaire (PSEQ) was used to assess the confidence people with knee pain have in performing activities while in pain.^1^ Higher scores of the PSEQ indicate higher levels of confidence to use the knee despite pain.^33^

### 2.4. Sample size

This study was considered a preliminary investigation, and no formal power calculation was performed. We planned to recruit between 12 and 15 participants per group based on the recommendation that preliminary studies for which little data exist to inform a formal sample size calculation should seek to recruit around 12 participants per group.^19^

### 2.5. Statistical analyses

Statistical analysis was performed using SPSS statistics ver.26 (IBM SPSS Statistics for MAC, Version 25.0; IBM Corp, Armonk, NY). A one-way analysis of variance and Fisher exact test were performed to test the differences in the sample characteristic between the groups. Analysis of covariance was conducted to assess group differences in clinical symptoms, adjusting for age, sex, pain duration, body mass index, K-L grade, and nonsteroidal anti-inflammatory drug use or nonuse. The Bonferroni method was performed for post hoc tests. To help with interpretation of the results from this preliminary investigation, effect sizes were calculated based on η^2^ (a large effect was defined as >0.14, a moderate effect as 0.06–0.14, and a small effect as <0.06).^7^ All *P* values were adjusted using the Benjamini–Hochberg procedure for multiple tests. False discovery rate–adjusted *P* values are reported.

## 3. Results

### 3.1. Sample characteristics

Sample characteristics are summarized in Table 1. Forty-six individuals participated in this study with 15 participants having both subjective and objective swelling (S + O group), 15 reporting subjective swelling in the absence of objective swelling (S only group), and 16 that had neither subjective nor objective swelling (No S/O group). No participants had evidence of objective swelling with no report of subjective swelling. There were no significant differences in sample characteristics between the 3 derived groups (Table 1).

**Table 1:** Sample characteristics.

	S only group (n = 15)	S + O group (n = 15)	No S/O group (n = 16)	Benjamini–Hochberg adjusted *P*
Mean age (y) (SD)	69.9 (10.4)	70.2 (7.4)	66.0 (8.1)	0.479
Gender (female/male)	15/0	14/1	12/4	0.360
Pain duration (wk)	4.5 (1.0–12.0)	2.5 (1.0–8.0)	4.9 (1.0–12.0)	0.361
BMI (SD)	26.0 (3.5)	25.5 (1.3)	25.1 (3.8)	0.704
Disease severity (K-L grade)				0.479
II	11	9	8	
III	1	3	6	
IV	3	3	2	
Medication (yes/no)	6/9	6/9	3/13	0.479

BMI, body mass index; K-L grade, Kellgren–Lawrence grade.

### 3.2. Clinical symptoms

Clinical symptoms are summarized in Table 2 (affected side) and Supplementary material 1 (available at http://links.lww.com/PR9/A135) (unaffected side).

**Table 2:** Comparison of clinical characteristics between groups.

	S only group (n = 15)	S + O group (n = 15)	No S/O group (n = 16)	Benjamini–Hochberg adjusted *P*	Effect size
Effusion area (cm^2^)	0.37 (0.17 to 0.78)*	1.47 (1.06 to 2.10)†‡	0.42 (0.10 to 0.95)*	<0.001	η^2^ = 0.62
FreKAQ (0–36)	18.3 (4.0 to 33.0)‡	19.0 (8.0 to 33.0)‡	4.4 (1.0 to 13.0)*†	<0.001	η^2^ = 0.30
Pain intensity (rest) (NRS 0–10)	2.7 (0 to 6.0)‡	2.3 (0 to 8.0)	0.4 (0 to 3.0)†	0.047	η^2^ = 0.12
Pain intensity (motion) (NRS 0–10)	6.2 (3.0 to 10.0)‡	5.1 (1 to 10.0)	4.7 (1.0 to 8.0)†	0.010	η^2^ = 0.15
Disability (OKS 0–48)	25.7 (14.0 to 40.0)‡	28.4 (18.0 to 43.0)‡	37.7 (26.0 to 43.0)*†	<0.001	η^2^ = 0.18
TPD threshold: medial (cm)	4.2 (1.0 to 17.5)*	1.7 (0.5 to 4.0)†	2.8 (0.5 to 7.5)	<0.001	η^2^ = 0.17
TPD threshold: lateral (cm)	4.5 (1.0 to 12.5)	2.9 (0.5 to 7.5)	2.6 (0.5 to 7.5)	0.064	η^2^ = 0.11
ROM: flexion (°)	129.4 (115.0 to 140.0)	133.3 (105.0 to 145.0)	137.2 (125.0 to 145.0)	0.061	η^2^ = 0.10
ROM: extension (°)	−8.0 (−15.0 to 0)	−8.3 (−15.0 to 0)	−8.4 (−10.0 to −5.0)	0.766	η^2^ = 0.01
Quadriceps muscle strength (N·m/kg)	10.7 (6.9 to 21.4)*‡	14.2 (1.7 to 33.8)†‡	18.9 (14.2 to 28.5)*†	<0.001	η^2^ = 0.17
PCS (0–52)	36.4 (21.0 to 45.0)*‡	24.9 (8.0 to 34.0)†‡	18.3 (8.0 to 27.0)*†	<0.001	η^2^ = 0.44
PSEQ (0–60)	32.3 (20.0 to 48.0)*‡	41.2 (25.0 to 53.0)†	44.4 (36.0 to 56.0)†	<0.001	η^2^ = 0.28

Effect sizes (η^2^: A large effect was defined as >0.14, a moderate effect as 0.06–0.14, and a small effect as <0.06).

* Differences are significant (*P* < 0.05) compared with the S + O only group.

† Differences are significant (*P* < 0.05) compared with the S only group.

‡ Differences are significant (*P* < 0.05) compared with the No S/O group.

FreKAQ, Fremantle Knee Awareness Questionnaire; NRS, Numeric Rating Scale; OKS, Oxford Knee Score; PCS, Pain Catastrophizing Scale; PSEQ, Pain Self-Efficacy Questionnaire; ROM, range of motion; TPD, 2-point discrimination.

#### 3.2.1. Extent of effusion area

As expected, the extent of the effusion area was significantly greater in the S + O group than in the S only group and the No S/O group (both, *P* < 0.05).

#### 3.2.2. Disturbed body perception

There was no significant difference in FreKAQ scores between the S only group and the S + O group, whereas scores in both these groups were significantly higher than the No S/O group (both, *P* < 0.05).

#### 3.2.3. Pain intensity

Both pain intensity at rest and pain intensity with movement were significantly higher in the S only group than in the No S/O group (*P* < 0.05).

#### 3.2.4. Disability

The Oxford Knee Score scores in the S only group and the S + O group were significantly lower (more disability) than those in the No S/O group (both, *P* < 0.05).

#### 3.2.5. Two-point discrimination threshold

No significant differences were found between the 3 groups for the TPD threshold at the lateral aspect of the knee (*P* = 0.06). Two-point discrimination threshold at the medial aspect of the knee was significantly larger in the S only group than in the S + O group (*P* < 0.05), indicative of reduced tactile acuity in the S only group.

#### 3.2.6. Range of motion

No significant differences between the 3 groups were found for knee flexion (*P* = 0.06) or knee extension ROM (*P* = 0.76).

#### 3.2.7. Quadriceps muscle strength

Quadriceps muscle strength was lowest in the S only group and was significantly less than both the S + O group and the No S/O group (both, *P* < 0.05). There was significant difference between the S + O group and No S/O group (*P* < 0.05).

#### 3.2.8. Pain catastrophizing

The Pain Catastrophizing Scale scores were highest (most dysfunctional) in the S only group and was significantly higher than both the S + O group and the No S/O group (both, *P* < 0.05). There was significant difference between the S + O group and No S/O group (*P* < 0.05).

#### 3.2.9. Pain self-efficacy

The PSEQ scores were lowest (most dysfunctional) in the S only group and were significantly lower than both the S + O group and the No S/O group (both, *P* < 0.05). There was no significant difference between the S + O group and No S/O group (*P* = 1.00).

## 4. Discussion

This is the first study to demonstrate that about 30% of people with knee OA report that their knee feels enlarged or swollen despite the absence of objective markers of knee swelling. We found that pain intensity and disability in those with only subjective reports of knee swelling was similar to those who had both subjective and objective swelling but more severe than those without any subjective or objective swelling. Those with only subjective reports of swelling had larger TPD distance threshold on the medial side of the knee than those who had both subjective and objective swelling and had greater levels of pain catastrophizing and poorer pain-related self-efficacy than both other groups. The tentative conclusion from these findings is that people who perceive their knees to be swollen in the absence of measurable effusion may be experiencing changes in how their knees are represented within the central nervous system (based on TPD findings) and have less confidence in using the knee and more maladaptive beliefs about the knee in pain.

A multicenter prospective study investigated the presence of effusion in the suprapatellar area in 600 people with chronic, painful, and primary knee OA and showed that joint effusion was present in 30% of this population.^9^ This is in close agreement with this study in which 33% of people had joint effusion. Some previous investigations have found a relationship between the presence of joint effusion and both pain^16,32^ and disability.^46^ This is consistent with the results reported here as we also found that people with objective signs of swelling (S + O group) reported higher levels of pain and disability than those with no effusion (No S/O group). However, there are data supporting a less clear relationship between joint effusion and pain,^14,64^ and it may be that the presence of a phenotype with high levels of pain, who only perceive the knee as swollen, contributes to this lack of a clear relationship. It is possible that a more in-depth evaluation of the relationship between objective and subjective swelling will help resolve these discrepancies in the literature.

The TPD threshold on the medial side of the knee was significantly larger for the subjective swelling only group than the group with both subjective and objective swelling. This finding is consistent with previous studies that showed that an expanded pictorial representation of the painful area in people with complex regional pain syndrome^41^ and low back pain^34^ was associated with increased TPD values. Some authors have suggested that the TPD threshold might serve as a simple clinical signature of reorganization within the somatosensory cortex specific to the body part tested.^4,23,30^ We know of no direct evidence of cortical changes in people with knee OA, but it is plausible that disruption of the brain grounded representation of the knee might be present in those who report disrupted conscious representation of the knee. Previous data support that increased TPD thresholds seem to be specific to the painful area,^11,42,62^ and the failure to detect any differences on the lateral side of the knee might reflect the greater prevalence of medial knee pain in knee OA,^61^ or be a reflection of the small sample size. Indeed, past work in knee OA has shown TPD impairments both medial and lateral to the patella.^50^ The small sample size, along with an analysis that corrected for multiple comparisons, might also contribute to the lack of observed difference in TPD threshold between the subjective swelling only group and the no subjective/objective swelling group. Clearly, further research is required.

Experimental studies have found that acute knee effusions induce quadriceps alpha motor neuron inhibition at the spinal cord^18,40,47^ and knee joint effusion can influence knee mechanics and muscle activity during gait in knee OA.^44^ Hence, although there are clear mechanisms that might explain muscle weakness in the presence of an actual joint effusion, we found quadriceps weakness to be greatest in the subjective swelling only group. The reasons for this result can only be speculated based on the data available, but may be a protective response to a body part that does not feel right, a manifestation of sensory dysfunction and potential cortical reorganization or related to the more dysfunctional cognitive appraisal of the painful knee. A self-reinforcing interaction of these factors is also plausible.^57^

Our results also showed that pain catastrophization and pain-related self-efficacy in the subjective swelling only group were significantly more impaired than both other groups. It is interesting that the confidence in the knee and appraisal of the threat to the knee in pain is greatest in those whose self-perception of the knee is most disrupted, ie, it feels swollen although it is not swollen. Cross-sectional studies such as this preclude suggesting any but the most speculative causal relationships; however, it is plausible that a knee that does not feel right is more likely to be appraised as untrustworthy and under threat. Longitudinal data that sequentially evaluates these constructs are required to further explore these issues.

There are a few limitations in this study. First, there are multiple methods available to evaluate perceived body size, such as questionnaires, template matching tasks in which people select from a range of images the one that best reflects their perception of their own body form, tasks in which participants are asked to complete drawings of how they perceive their body, or digital equivalents in which on-screen representations of the body are manipulated to match the perceived body size. However, there is still no golden standard method; thus, the criterion-related validity of the question used in this study is currently unknown. Second, the sample size is relatively small, which may have affected the results, as discussed above. Third, only 5 of the 46 participants in this study were male (11%), which does not reflect the typical gender balance in the knee OA population.^48^ This may impact on the generalizability of these findings. Fourth, x-ray was used to estimate OA severity by KL grades; thus, our results our limited to pathology seen on x-ray. It is possible that more sensitive imaging methods (such as computed tomography or MRI) might detect further detail, although this comes with similar limitations given recent work showing that the prevalence of knee OA features on MRI is high even in uninjured, asymptomatic people.^8^ Fifth, the study was cross-sectional; so, any causal relationship between pain, disability, tactile acuity, muscle strength, maladaptive belief, and expanded body image is unknown and beyond the scope of this article. Hence, a longitudinal study is needed to explore causality and the temporal sequencing of these findings.

There are potentially relevant clinical implications from our findings. In recent years, it has been suggested that improved clinical outcomes may occur if individualized treatment, based on relevant clinical phenotypes, is undertaken. For example, this has underscored recommendations in back pain care, including the use of the STarT Back Screening Tool^17^ or Örebro Musculoskeletal Pain Questionnaire.^22^ Here, we have provided preliminary evidence that a portion of people with knee OA experience perceptions of knee swelling in the absence of objective markers of swelling. Past work in therapeutic targeting of body perception has shown that visuotactile illusions applied using mediated reality can alter the perceived size of the knee in people with knee OA and that such perceptual shifts are analgesic.^49^ Intriguingly, these illusions can also alter objective knee swelling in some cases,^24^ making them an interesting method to explore whether the phenotypes identified here might have differing clinical responses to brain-targeted treatment. Furthermore, the group reporting only subjective feelings of swelling also had impaired tactile acuity, raising the possibility that treatment targeting impaired sensorimotor representation (such as touch discrimination training and implicit motor imagery training) may be most relevant in this group. Indeed, failure of such treatments to improve pain relief or knee function in people with knee OA in past work^15^ may well represent a mismatch between the patient (eg, lack of sensory impairment) and the treatment. Further work to explore whether this group of people with knee OA who have perceptual impairments regarding the painful body part respond differently to treatment than those without perceptual dysfunction is warranted.

## 5. Conclusion

Our results show that some people with knee pain experience subjective feelings of knee swelling without any evidence of objective swelling detected by ultrasonography and that this group has severe pain and functional disability. Furthermore, these people seem to have poorer tactile acuity, decreased muscle strength, and more dysfunctional beliefs about the knee in pain. Longitudinal data are needed to further understand how these factors interact. Specific exploration of altered perception of the knee might be useful in people with knee OA and targeting any maladaptive size perception may be a potential treatment target for this group.

## Disclosures

T. R. Stanton is supported by a National Health & Medical Research Council Career Development Fellowship (ID1141735). The remaining authors have no conflicts of interest to declare. The authors alone are responsible for the contents and writing up of our study.

## Appendix A. Supplemental digital content

Supplemental digital content associated with this article can be found online at http://links.lww.com/PR9/A135.

## Supplementary Material

SUPPLEMENTARY MATERIAL

## References

[1] Adachi T Nakae A Maruo T Shi K Shibata M Maeda L Saitoh Y Sasaki J. Validation of the Japanese version of the pain self-efficacy questionnaire in Japanese patients with chronic pain. Pain Med 2014;15:1405–17. 24717053 10.1111/pme.12446

[2] Altman R Alarcón G Appelrouth D Bloch D Borenstein D Brandt K Brown C Cooke TD Daniel W Feldman D Greenwald R Hochberg M Howell D Ike R Kapila P Kaplan D Koopman W Marino C Mcdonald E Mcshane DJ Medsger T Michel B Murphy WA Osial T Ramsey-Goldman R Rothschild B Wolfe F. The American College of Rheumatology criteria for the classification and reporting of osteoarthritis of the hip. Arthritis Rheum 1991;34:505–14. 2025304 10.1002/art.1780340502

[3] Brosseau L Tousignant M Budd J Chartier N Duciaume L Plamondon S O'Sullivan JP O'Donoghue S Balmer S. Intratester and intertester reliability and criterion validity of the parallelogram and universal goniometers for active knee flexion in healthy subjects. Physiother Res Int 1997;2:150–66. 9421820 10.1002/pri.97

[4] Catley MJ Tabor A Wand BM Moseley GL. Assessing tactile acuity in rheumatology and musculoskeletal medicine—how reliable are two-point discrimination tests at the neck, hand, back and foot? Rheumatol 2013;52:1454–61. 10.1093/rheumatology/ket140 23611918

[5] Chiba D Ota S Sasaki E Tsuda E Nakaji S Ishibashi Y. Knee effusion evaluated by ultrasonography warns knee osteoarthritis patients to develop their muscle atrophy: a three-year cohort study. Sci Rep 2020;10:8444. 32439881 10.1038/s41598-020-65368-4 PMC7242413

[6] Chiba D Tsuda E Maeda S Sasaki E Takahashi I Nakaji S Ishibashi Y. Evaluation of a quantitative measurement of suprapatellar effusion by ultrasonography and its association with symptoms of radiographic knee osteoarthritis: a cross-sectional observational study. Arthritis Res Ther 2016;18:181–8. 27487832 10.1186/s13075-016-1078-y PMC4973041

[7] Cohen J. Statistical power analysis for the behavioral sciences. 2nd edn. Hillsdale: Lawrence Erlbaum, 1988.

[8] Culvenor AG Øiestad BE Hart HF Stefanik JJ Guermazi A Crossley KM. Prevalence of knee osteoarthritis features on magnetic resonance imaging in asymptomatic uninjured adults: a systematic review and meta-analysis. Br J Sports Med 2019;53:1268–78. 29886437 10.1136/bjsports-2018-099257 PMC6837253

[9] D'Agostino MA Conaghan P LeBars ML Baron G Grassi W Martin-Mola E Wakefield R Brasseur J-L So A Bachhaus M Malaise M Burmester G Schmidely N Ravaud P Dougados M Emery P. EULAR report on the use of ultrasonography in painful knee osteoarthritis. Part 1: prevalence of inflammation in osteoarthritis. Ann Rheum Dis 2005;64:1703–9. 15878903 10.1136/ard.2005.037994 PMC1755310

[10] Dawson J Fitzpatrick R Murray D Carr A. Questionnaire on the perceptions of patients about total knee replacement. J Bone Joint Surg Br 1998;80:63–9. 9460955 10.1302/0301-620x.80b1.7859

[11] Debenham J Butler P Mallows A Wand BM. Disrupted tactile acuity in people with achilles tendinopathy: a preliminary case-control investigation. J Orthop Sports Phys Ther 2016;46:1061–4. 27796191 10.2519/jospt.2016.6514

[12] Eşen S Akarırmak U Aydın FY Unalan H. Clinical evaluation during the acute exacerbation of knee osteoarthritis: the impact of diagnostic ultrasonography. Rheumatol Int 2013;33:711–7. 22562715 10.1007/s00296-012-2441-1

[13] Fransen M Crosbie J Edmonds J. Isometric muscle force measurement for clinicians treating patients with osteoarthritis of the knee. Arthritis Rheum 2003;49:29–35. 12579591 10.1002/art.10923

[14] Hall M Doherty S Courtney P Latief K Zhang W Doherty M. Synovial pathology detected on ultrasound correlates with the severity of radiographic knee osteoarthritis more than with symptoms. Osteoarthritis Cartilage 2014;22:1627–33. 25278071 10.1016/j.joca.2014.05.025 PMC4192137

[15] Harms A Heredia-Rizo AM Moseley GL Hau R Stanton TR. A feasibility study of brain-targeted treatment for people with painful knee osteoarthritis in tertiary care. Physiother Theor Pract 2020;36:142–56. 10.1080/09593985.2018.1482391 29889597

[16] Hill CL Gale DG Chaisson CE Skinner K Kazis L Gale ME Felson DT. Knee effusions, popliteal cysts, and synovial thickening: association with knee pain in osteoarthritis. J Rheumatol 2001;28:1330–7. 11409127

[17] Hill JC Whitehurst DG Lewis M Bryan S Dunn KM Foster NE Konstantinou K Main CJ Mason E Somerville S Sowden G Vohora K Hay EM. Comparison of stratified primary care management for low back pain with current best practice (STarT Back): a randomised controlled trial. Lancet 2011;378:1560–71. 21963002 10.1016/S0140-6736(11)60937-9 PMC3208163

[18] Hopkins JT Ingersoll CD Krause BA Edwards JE Cordova ML. Effect of knee joint effusion on quadriceps and soleus motoneuron pool excitability. Med Sci Sports Exerc 2001;33:123–6. 11194097 10.1097/00005768-200101000-00019

[19] Julious SA. Sample size of 12 per group rule of thumb for a pilot study. Pharm Stat 2005;4:287–91.

[20] Kellgren JH Lawrence JS. Radiological assessment of osteo-arthrosis. Ann Rheum Dis 1957;16:494–502. 13498604 10.1136/ard.16.4.494 PMC1006995

[21] Lewis JS Kersten P McCabe CS McPherson KM Blake DR. Body perception disturbance: a contribution to pain in complex regional pain syndrome (CRPS). PAIN 2007;133:111–9. 17509761 10.1016/j.pain.2007.03.013

[22] Linton SJ Boersma K. Early identification of patients at risk of developing a persistent back problem: the predictive validity of the Örebro Musculoskeletal Pain Questionnaire. Clin J Pain 2003;19:80–6. 12616177 10.1097/00002508-200303000-00002

[23] Lotze M Moseley GL. Role of distorted body image in pain. Curr Rheumatol Rep 2007;9:488–96. 18177603 10.1007/s11926-007-0079-x

[24] MacIntyre E Sigerseth M Pulling BW Newport R Stanton TR. The effect of knee resizing illusions on pain and swelling in symptomatic knee osteoarthritis: a case report. Pain Rep 2019;4:e795. 31984300 10.1097/PR9.0000000000000795 PMC6903346

[25] Mancini F Longo MR Iannetti GD Haggard P. A supramodal representation of the body surface. Neuropsychologia 2011;49:1194–201. 21199662 10.1016/j.neuropsychologia.2010.12.040

[26] Matsuoka H Sakano Y. Assessment of cognitive aspect of pain: development, reliability, and validation of Japanese version of pain catastrophizing scale. Jpn J Psychosom Med 2007;47:95–102.

[27] Moberg E. Two-point discrimination test. A valuable part of hand surgical rehabilitation, e.g. in tetraplegia. Scand J Rehabil Med 1990;22:127–34. 2244189

[28] Monticone M Sconza C Portoghese I Nishigami T Wand BM Sorrentino G Lemorini G Respizzi S Giordano A Franchignoni F. Cross-cultural adaptation, reliability and validity of the Fremantle Knee Awareness Questionnaire in Italian subjects with painful knee osteoarthritis. Health Qual Life Outcomes 2021;19:1–10. 33827594 10.1186/s12955-021-01754-4 PMC8025485

[29] Moreira C Bassi AR Brandão MP Silva AG. Do patients with chronic neck pain have distorted body image and tactile dysfunction? Eur J Physiother 2017;19:215–21.

[30] Moseley GL Flor H. Targeting cortical representations in the treatment of chronic pain: a review. Neurorehabil Neural Repair 2012;26:646–52. 22331213 10.1177/1545968311433209

[31] Moseley GL Parsons TJ Spence C. Visual distortion of a limb modulates the pain and swelling evoked by movement. Curr Biol 2008;18:R1047–8. 19036329 10.1016/j.cub.2008.09.031

[32] Naredo E Cabero F Palop MJ Collado P Cruz A Crespo M. Ultrasonographic findings in knee osteoarthritis: a comparative study with clinical and radiographic assessment. Osteoarthritis Cartilage 2005;13:568–74. 15979008 10.1016/j.joca.2005.02.008

[33] Nicholas MK. The pain self‐efficacy questionnaire: taking pain into account. Eur J Pain 2007;11:153–63. 16446108 10.1016/j.ejpain.2005.12.008

[34] Nishigami T Mibu A Osumi M Son K Yamamoto S Kajiwara S Tanaka K Matsuya A Tanabe A. Are tactile acuity and clinical symptoms related to differences in perceived body image in patients with chronic nonspecific lower back pain? Man Ther 2015;20:63–7. 25081221 10.1016/j.math.2014.06.010

[35] Nishigami T Mibu A Tanaka K Yamashita Y Yamada E Wand BM Catley MJ Stanton TR Moseley GL. Development and psychometric properties of knee-specific body-perception questionnaire in people with knee osteoarthritis: the Fremantle Knee Awareness Questionnaire. PLoS One 2017;12:e0179225. 28650969 10.1371/journal.pone.0179225 PMC5484477

[36] Nishigami T Mibu A Tanaka K Yamashita Y Shimizu ME Wand BM Catley MJ Stanton TR Moseley GL. Validation of the Japanese version of the Fremantle Back Awareness Questionnaire in patients with low back pain. Pain Pract 2018;18:170–9. 28422409 10.1111/papr.12586

[37] Nishigami T Tanaka S Mibu A Imai R Wand BM. Knee-related disability was largely influenced by cognitive factors and disturbed body perception in knee osteoarthritis. Sci Rep 2021;11:5835–7. 33712725 10.1038/s41598-021-85307-1 PMC7970993

[38] Nishigami T Watanabe A Maitani T Shigetoh H Mibu A Wand BM Catley MJ Stanton TR Moseley GL. Development and validation of a shoulder-specific body-perception questionnaire in people with persistent shoulder pain. BMC Musculoskelet Disord 2021;22:98–11. 33478446 10.1186/s12891-021-03944-z PMC7819341

[39] Nordesjö LO Nordgren B Wigren A Kolstad K. Isometric strength and endurance in patients with severe rheumatoid arthritis or osteoarthrosis in the knee joints. Scand J Rheumatol 1983;12:152–6. 6857173 10.3109/03009748309102902

[40] Palmieri RM Weltman A Edwards JE Tom JA Saliba EN Mistry DJ Ingersoll CD. Pre-synaptic modulation of quadriceps arthrogenic muscle inhibition. Knee Surg Sports Traumatol Arthrosc 2005;13:370–6. 15685462 10.1007/s00167-004-0547-z

[41] Peltz E Seifert F Lanz S Müller R Maihöfner C. Impaired hand size estimation in CRPS. J Pain 2011;12:1095–101. 21741321 10.1016/j.jpain.2011.05.001

[42] Peters ML Schmidt AJ. A comparison of two-point discrimination threshold of tactual, non-painful stimuli between chronic low back pain patients and controls. PAIN 1991;44:57–60. 1828112 10.1016/0304-3959(91)90147-P

[43] Pleger B Foerster AF Ragert P Dinse HR Schwenkreis P Malin JP Nicolas V Tegenthoff M. Functional imaging of perceptual learning in human primary and secondary somatosensory cortex. Neuron 2003;40:643–53. 14642286 10.1016/s0896-6273(03)00677-9

[44] Rutherford DJ Hubley-Kozey CL Stanish WD. Knee effusion affects knee mechanics and muscle activity during gait in individuals with knee osteoarthritis. Osteoarthritis Cartilage 2012;20:974–81. 22698444 10.1016/j.joca.2012.05.014

[45] Senkowski D Heinz A. Chronic pain and distorted body image: implications for multisensory feedback interventions. Neurosci Biobehav Rev 2016;69:252–9. 27524638 10.1016/j.neubiorev.2016.08.009

[46] Sowers M Karvonen-Gutierrez CA Jacobson JA Jiang Y Yosef M. Associations of anatomical measures from MRI with radiographically defined knee osteoarthritis score, pain, and physical functioning. J Bone Joint Surg Am 2011;93:241–51. 21266638 10.2106/JBJS.I.00667 PMC3028452

[47] Spencer JD Hayes KC Alexander IJ. Knee joint effusion and quadriceps reflex inhibition in man. Arch Phys Med Rehabil 1984;65:171–7. 6712434

[48] Srikanth VK Fryer JL Zhai G Winzenberg TM Hosmer D Jones G. A meta-analysis of sex differences prevalence, incidence and severity of osteoarthritis. Osteoarthritis Cartilage 2005;13:769–81. 15978850 10.1016/j.joca.2005.04.014

[49] Stanton TR Gilpin HR Edwards L Moseley GL Newport R. Illusory resizing of the painful knee is analgesic in symptomatic knee osteoarthritis. PeerJ 2018;6:e5206. 30038863 10.7717/peerj.5206 PMC6054060

[50] Stanton TR Lin CW Bray H Smeets RJ Taylor D Law RY Moseley GL. Tactile acuity is disrupted in osteoarthritis but is unrelated to disruptions in motor imagery performance. Rheumatology (Oxford) 2013;52:1509–19. 23661429 10.1093/rheumatology/ket139

[51] Sullivan MJ Bishop SR Pivik J. The pain catastrophizing scale: development and validation. Psychol Assess 1995;7:524.

[52] Sündermann O Flink I Linton SJ. My body is not working right: a cognitive behavioral model of body image and chronic pain. PAIN 2020;161:1136–9. 32028333 10.1097/j.pain.0000000000001822

[53] Takeuchi R Sawaguchi T Nakamura N Ishikawa H Saito T Goldhahn S. Cross-cultural adaptation and validation of the Oxford 12-item knee score in Japanese. Arch Orthop Trauma Surg 2011;131:247–54. 20830479 10.1007/s00402-010-1185-1

[54] Tanaka S Nishigami T Wand BM Stanton TR Mibu A Tokunaga M Yoshimoto T Ushida T. Identifying participants with knee osteoarthritis likely to benefit from physical therapy education and exercise: a hypothesis‐generating study. Eur J Pain 2021;25:485–96. 33108042 10.1002/ejp.1687

[55] van Selm MJ Gibson WI Travers MJ Moseley GL Hince D Wand BM. Visually induced analgesia in a deep tissue experimental pain model: a randomised crossover experiment. Eur J Pain 2018;22:1448–56. 10.1002/ejp.1234 29676836

[56] Viceconti A Camerone EM Luzzi D Pentassuglia D Pardini M Ristori D Rossettini G Gallace A Longo MR Testa M. Explicit and implicit own's body and space perception in painful musculoskeletal disorders and rheumatic diseases: a systematic scoping review. Front Hum Neurosci 2020;14:83. 32327984 10.3389/fnhum.2020.00083 PMC7161420

[57] Wand BM. Chronic low back pain: a maladaptive perceptions model. NOI 2012: neurodynamics and the neuromatrix conference. Adelaide, Australia, 2012. Available at: (https://researchonline.nd.edu.au/cgi/viewcontent.cgi?article=1010&context=physiotherapy_conference&fbclid=IwAR1k3UoRD6A2825-NEBujp1OuSbMsyvAKj3v3waZ2MkKU4Wz3SLGtRJdXgc).

[58] Wand BM Catley MJ Rabey MI O'Sullivan PB O'connell NE Smith AJ. Disrupted self-perception in people with chronic low back pain. Further evaluation of the Fremantle Back Awareness Questionnaire. J Pain 2016;17:1001–12. 27327235 10.1016/j.jpain.2016.06.003

[59] Watkins MA Riddle DL Lamb RL Personius WJ. Reliability of goniometric measurements and visual estimates of knee range of motion obtained in a clinical setting. Phys Ther 1991;71:90–7. 1989012 10.1093/ptj/71.2.90

[60] Wittkopf PG Lloyd DM Johnson MI. Changing the size of a mirror‐reflected hand does not affect pain perception: a repeated measures study on healthy human participants. Eur J Pain 2018;22:527–37. 29082635 10.1002/ejp.1135

[61] Wood LRJ Peat G Thomas E Duncan R. Knee osteoarthritis in community-dwelling older adults: are there characteristic patterns of pain location? Osteoarthr Cartilage 2007;15:615–23. 10.1016/j.joca.2006.12.001 17276094

[62] Yamashita H Nishigami T Mibu A Tanaka K Manfuku M Fukuhara H Yoshino K Seto Y Wand BM. Perceived body distortion rather than actual body distortion is associated with chronic low back pain in adults with cerebral palsy: a preliminary investigation. Pain Pract 2019;19:826–35. 31264357 10.1111/papr.12815

[63] Yamashita Y Nishigami T Mibu A Tanaka K Wand BM Catley MJ Higashi T. Development and psychometric testing of the Japanese version of the Fremantle neck awareness questionnaire: a cross-sectional study. J Pain Res 2021;4:311–24. 10.2147/JPR.S267930 PMC7870290 33568938

[64] Zhang Y Nevitt M Niu J Lewis C Torner J Guermazi A Roemer F McCulloch C Felson DT. Fluctuation of knee pain and changes in bone marrow lesions, effusions, and synovitis on magnetic resonance imaging. Arthritis Rheum 2011;63:691–9. 21360498 10.1002/art.30148 PMC3056156

