# Supplementary material for: “But it feels swollen!”: the frequency and clinical characteristics of people with knee osteoarthritis who report subjective knee swelling in the absence of objective swelling
Source: Pain Rep. 2021 Nov 8;6(4):e971. doi: 10.1097/PR9.0000000000000971 (PMC8577815; doi:10.1097/PR9.0000000000000971)
Supplement: SUPPLEMENTARY MATERIAL [file painreports-6-e971-s001.pdf]

Supplementary material 1. Unaffected side data

	S only group (n=15)	S+O group (n=15)	No S/O group (n=16)
Effusion area (cm^2)	0.36 (0.21 to 0.65)	0.41 (0.20 to 0.82)	0.35 (0.19 to 0.71)
NRS (rest) (0-10)	0.2 (0 to1)	0.1 (0 to 1)	0.06 (0 to 1)
NRS (motion) (0-10)	0.47 (0 to 2)	0.8 (0 to 3)	0.38 (0 to 2)
TPD threshold: Medial (cm)	2.1 (0.5 to 5.5)	1.5 (0.5 to 3.5)	2.6 (1.0 to 7.0)
TPD threshold: Lateral (cm)	2.0 (0.5 to 4.5)	2.4 (0.5 to 4.5)	2.6 (1.0 to 7.0)
ROM: Flexion (°)	137.3 (120.0 to 145.0)	138.7 (110.0 to 145.0)	138.8 (130.0 to 145.0)
ROM: Extension (°)	-3.3 (-10.0 to 0)	-3.7 (-10.0 to 0)	-2.8 (-10.0 to 0)
Quadriceps muscle strength (Nm/kg)	16.7 (10.7 to 27.8)	18.7 (8.6 to 32.7)	19.2 (11.7 to 28.5)

NRS, numeric rating scale; ROM, range of motion; TPD, 2-point discrimination; US, ultrasound; OKS, Oxford knee score; PCS, Pain Catastrophizing Scale; PSEQ, Pain Self Efficacy Questionnaire; FreKAQ, Fremantle Knee Awareness Questionnaire
